# Comparison of Universal mtDNA Primers in Species Identification of Animals in a Sample with Severely Degraded DNA

**DOI:** 10.3390/ani14223256

**Published:** 2024-11-13

**Authors:** Aleksandra Figura, Magdalena Gryzinska, Andrzej Jakubczak

**Affiliations:** Institute of Biological Basis of Animal Production, University of Life Sciences in Lublin, 20-950 Lublin, Poland; magdalena.gryzinska@up.lublin.pl (M.G.); andrzej.jakubczak@up.lublin.pl (A.J.)

**Keywords:** mitochondrial DNA, DNA barcoding, cytochrome b, 12S rRNA, 16S rRNA, species identification

## Abstract

Analysis of mitochondrial DNA, specifically the cytochrome b gene (cyt b), has become an essential tool for species identification. In the case of degraded samples, in which DNA is fractionated, universal primers, which are highly effective at amplifying the target region, are necessary. The material analysed in this study was a keychain made of bone, which was secured at a border crossing due to the suspicion that it was made of ivory. Due to processing of the bone and the likelihood of DNA degradation, five pairs of universal primers with different product lengths (from 148 to 990 base pairs) were used for species identification. Fragments of mtDNA from the cyt b and the 12S rRNA and 16S rRNA subunits were analysed. The analysis showed that only one pair of primers (L15601/H15748) enabled identification of the species, which is very common in samples with highly degraded DNA. The material was bone tissue belonging to the species *Bos taurus* (cattle). Species identification by molecular methods is extremely important in analysis of material when the species cannot be identified on the basis of morphological characteristics.

## Simple Summary

Genetic methods can currently be used to identify the species of an animal on the basis of the animal itself, its fragments or tissues, or products obtained from it. This study presents typical molecular markers used in species identification. It presents a procedure comparing mtDNA primers used to determine the species of animal from which an item secured at a border crossing (a keychain) was made.

## 1. Introduction

DNA testing plays an important role in cases of identification. Nuclear DNA (nDNA) is routinely used for individual identification, while mitochondrial DNA (mtDNA) is used for species identification. Inheritance of mtDNA differs from inheritance of nDNA, which consists of X and Y sex chromosomes as well as autosomes from the father and mother, whereas mtDNA is inherited only from the mother. The number of copies of mitochondrial DNA in a cell ranges from 1000 to 10,000 [1]. When the biological material being tested is highly degraded, the large number of copies of mitochondrial DNA in the cell increases the probability that the analysis will be successful [2]. DNA degradation, caused by environmental factors and the passage of time, leads to the fragmentation of molecules and the loss of sequence integrity, which can significantly hinder effective genetic analysis, especially in the case of samples that have not been stored under appropriate conditions. As a result of degradation, genetic analyses are often limited to shorter DNA fragments that are more resistant to degradation processes than longer sequences. Therefore, the use of markers with small amplicons, such as mini-barcodes, is crucial for accurate species identification in highly degraded material. Studies show that DNA degradation can limit amplification and genetic analysis, highlighting the importance of selecting appropriate genetic markers and analytical methods [3]. The mitochondrial genome is widely used in phylogenetic analyses, because it evolves much more rapidly than nuclear DNA, causing differences to accumulate in closely related species [4].

Among farm animals, mtDNA sequences were first determined for cattle [5], and then for horses [6], sheep and pigs [7,8], and later for goats [9]. The sequences of the first mitogenomes of extinct bird species were obtained for the bush moa (*Anomalopteryx didiformis*) and Eastern moa (*Emeus crassus*) [10,11], which made it possible to explain the phylogenetic relationships between these extinct species and the largest extant flightless birds of the order Struthioniformes [12].

Two main types of primers are used in PCR for species identification. Specific primers bind to the target sequence in the genome of a given species and are characteristic of that species alone (species-specific). They are specially designed and require prior knowledge of the species under analysis. The PCR result confirms or rules out a given species. Universal primers, on the other hand, are designed for a group of organisms (e.g., vertebrates), and the result indicates a specific species or related species.

There are 5–12 million species in the world, of which only 1.7 million have been identified. They are mainly distinguished on the basis of morphological characters, which requires a great deal of labour and specialised knowledge [13]. DNA barcoding is a method involving sequencing of one or several loci for species identification. The use of a very short, defined genome sequence makes it possible to obtain a ‘DNA barcode’—an image of the sequence of bases in a section of DNA. DNA barcodes can be compared to determine the species of individuals [13,14]. DNA barcoding is a basic tool for discovering, describing, and understanding biological diversity, and specifically for species identification. At first it was expected that a single genetic marker would serve as a universal DNA barcode. In practice, however, various regions of DNA are necessary to ensure correct species identification [15].

This led to the search for a section of DNA that could be used to distinguish species at different stages of development. In the animal kingdom, mitochondrial genes are often the preferred choice for species identification because they offer several advantages over nuclear genes. These advantages include the absence of introns, the low probability of recombination, the presence of conserved primer sites, and a simple, defined sequence. These features make mitochondrial genes, including those encoding cytochrome subunits, ideal markers for animal identification. In addition, mitochondrial genes are present in many copies, in contrast to genes from the nuclear genome [13].

Universal pairs of primers are used to amplify the region of the gene encoding cytochrome c oxidase subunit I (COI). By amplifying the same gene in different organisms, it is possible to create a library of gene sequences. It is essential to know the taxonomic group of the organism of interest (fish, birds, mammals, etc.), because the primers used in PCR are specific for taxonomic groups [16]. Amplification is followed by sequencing of the PCR product. There are basic databases for DNA sequence analysis: the Barcode of Life Data Systems (BOLD), the National Center for Biotechnology Information (NCBI), the European Molecular Biology Laboratory’s European Bioinformatics Institute (EMBL-EBI), and the DNA Data Bank of Japan (DDBJ). Owing to the similarity search algorithm of the Basic Local Alignment Search Tool (BLAST), it is possible to compare DNA sequences to the data contained in the available databases and with one another.

The BOLD database is a publicly available tool that compares a DNA sequence to samples that have already been identified and contains additional data about the sample. This database is a centre of information about DNA barcodes [16]. It can be used to find taxonomic information, i.e., a dendrogram of individual taxa, and also to determine the genetic distance between taxonomic units. The BOLD database is a library containing primarily the DNA sequences of animals. It operates based on the gene encoding cytochrome c oxidase (COI). NCBI BLAST is a publicly available online tool used for identification of an unknown sequence [16].

Universal primers L1085/H1259 targeting the 12S rRNA and 16S rRNA genes are used for species identification of vertebrates [17]. The 12S rRNA gene has been used to identify the species of big cats such as jaguars, pumas, and ocelots [18], 38 species based on analysis of meat in northern Uganda [19], and 12 species of wild animals in a protected area in Lebanon [20]. In the case of 16S rRNA, primers L2513 and H2714 have been used to confirm species such as jaguars and pumas in protected areas in Costa Rica [21] and 12 different mammalian species in eastern China [22].

The aim of the study was to test five pairs of universal primers in PCR of highly degraded material in order to determine the species of animal from which a keychain secured at an air border crossing had been made, and whether this species was subject to protection under the Habitats Directive (92/43/EEC) [23] and the Washington Convention (CITES, 1973) [24].

## 2. Materials and Methods

### 2.1. Animals

**Figure 1:**
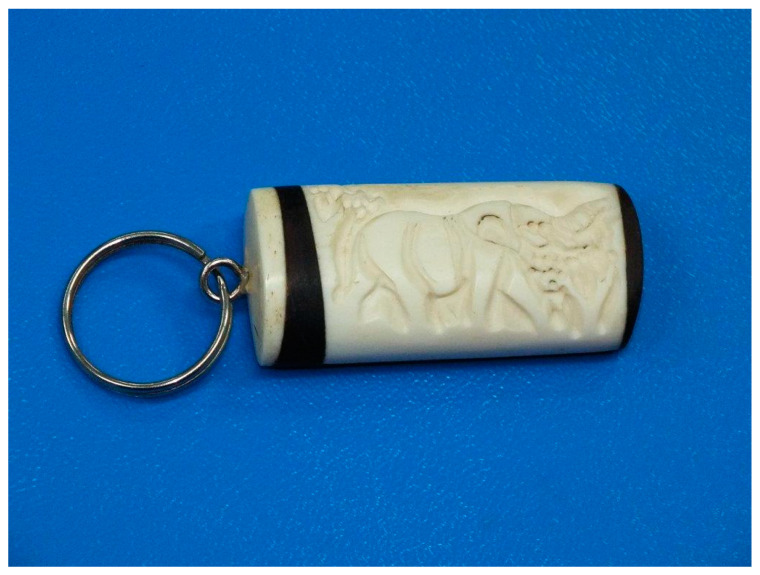
Material for analysis.

The material for the study was a keychain made of bone, delivered to the laboratory by the Customs Department of Lublin Airport (Figure 1). The keychain had been secured due to the suspicion that it might be made of ivory. Elephants are protected under legal provisions.

### 2.2. DNA Extraction, PCR Amplification, and Sequencing

Prior to DNA isolation, the material was kept at room temperature. Before being crushed, the bone (keychain) was cleaned, which was an important factor preventing simultaneous extraction of inhibitors and contaminants. The samples were cleaned with nuclease-free water to remove contaminants and then washed with 70% alcohol to speed up the drying process. Two holes were drilled in the bone using a prosthetic micromotor (ULTIMATE XL-GT) with dental drill bits (ball and conical), at a rotation speed of up to 50,000 rpm. In this way, 50 mg of fine bone powder (FBP) was obtained from each hole. This manner of obtaining the sample minimised destruction of the test material.

DNA was isolated from the bone using the PrepFiler^®^BTA Forensic DNA Extraction Kit (Carlsbad, CA, USA). The material obtained was subjected to lysis on a thermal shaker at 56 °C for 120 min and to digestion using Proteinase K, followed by binding of DNA to magnetic beads. Next, isopropanol was added and the sample was purified using wash buffers. The isolated DNA was suspended in 50 µL of PrepFiler^®^ Elution Buffer. Following DNA extraction, the samples were stored at −20 °C.

The purity and concentration of isolated DNA were assessed by spectrophotometry in the BioPhotometer (Eppendorf, Hamburg, Germany) at wavelengths of 230 nm, 260 nm, 280 nm, and 320 nm. For the qualitative assessment, electrophoretic separation was carried out in a 1% agarose gel with SimplySafe ethidium bromide alternative (EURx) in 1 × TBE buffer, at 70 V for 40 min. The samples were visualised under UV light and archived using Scion Image v. 4.0.2 software.

PCR was carried out using five pairs of universal primers which are used for species identification (Table 1). Fragments of mtDNA from the cytochrome b gene (cyt b) and the 12S rRNA and 16S rRNA subunits were analysed. Primers for the cyt b and subunits 12S and 16S rRNA were selected on the basis of the available literature.

The PCR was optimised by choosing the optimal primer annealing temperature (50–60 °C) and modifying the magnesium cations which are a cofactor for DNA polymerase in the SensoQuest Labcycler thermal cycler (Syngen, Göttingen, Germany). PCR amplification was performed in a 25 μL reaction volume containing 5 μL of DNA (1 ng/μL), 1.0 U Taq polymerase (AmpliTaq Gold 360 DNA Polymerase, Applied Biosystems, Waltham, MA, USA) in the manufacturer’s buffer, adjusted to a final concentration of 2.5 mM MgCl_2_, 0.2 mM of each dNTP, and 0.1 mM of each primer. With each run, negative and positive PCR controls were performed for PCR validation. PCR cycling conditions were 95 °C for 7 min, 36 cycles of 95 °C for 45 s, 53 °C for 45 s, and 72 °C for 45 s, and a final extension step of 7 min at 72 °C (Labcycler, SensoQuest, Göttingen, Germany). The product was subjected to electrophoresis in a 2% agarose gel containing 0.01% SimplySafe. Electrophoresis was carried out at 90 V for 120 min.

The PCR products were sequenced by Sanger’s method using fluorescence-labelled dideoxynucleotides. PCR products were purified to remove unbound primers and dNTPs using the ExoSAP-IT kit (Affymetrix, Santa Clara, CA, USA). Sequencing PCR was performed using the BigDye^®^ Terminator v3.1 Cycle Sequencing Kit (Applied Biosystems) in a SensoQuest thermal cycler (Labcycler). The final volume of the reaction sample was 20 µL. Primers were diluted to a concentration of 3.2 pmol/µL. The primers used for sequencing PCR had the same sequence as those used for preparative PCR. For each sample, a reaction was carried out with the F and R primers for the purpose of assembling contigs at further stages of bioinformatics analysis. The thermal profile and composition of the reaction mix for sequencing PCR were optimised. Sequencing PCR products were purified using the ExTerminator kit (A&A Biotechnology, Gdańsk, Poland). DNA sequencing was performed in the 3100 Avant Genetic Analyzer (Applied Biosystems).

### 2.3. Data Analyses

The results of sequencing of the fragments were verified using DNA Baser software (DNA Baser Sequence Assembler v. 3). The sequences obtained following the initial analysis were the basis for further analysis. Contigs were assembled in the CAP3 program. The resulting nucleotide sequences were compared with results in the NCBI database using the BLAST algorithm.

## 3. Results

Biological material from bone tissue was analysed in the study in order to identify its species. Identification was based on species-specific molecular markers: the cytochrome b gene and the gene encoding ribosomal subunits 16S rRNA and 12S rRNA. Primers with product amplification lengths of 148, 306, and 990 base pairs were used for cyt b, and lengths of 215 and 244 base pairs for rRNA. Among the DNA fragments analysed, the bioinformatics analysis yielded results only for the shortest sequence, from the cyt b, which was amplified using the primer pair L15601 and H15748. These universal primers, with a short amplicon of 148 bp, have the advantage of enabling identification even of postmortem samples showing signs of degradation, and the short amplicon increases the chance of obtaining a product and, at the same time, reduces the risk of amplifying a fragment of contaminating exogenous DNA.

**Figure 2:**
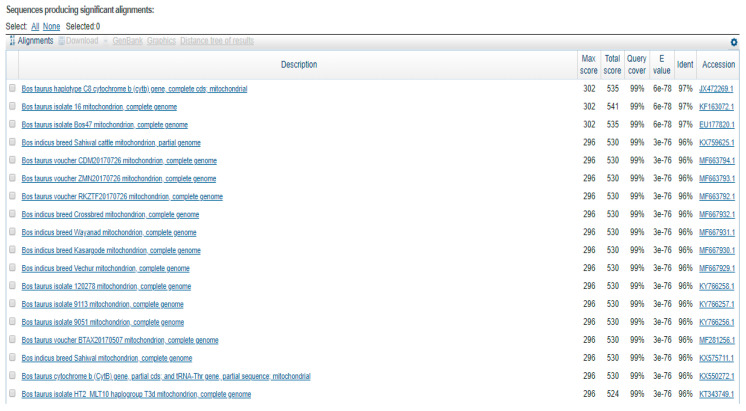
Results of the search for species similarity based on the sequences analysed (using BLAST)—list of sequences found, with percentage.

The analysis showed that according to taxonomic classification, the samples belong to the species *Bos taurus* (domestic cattle) (Figure 2). The comparative analysis revealed a high degree of similarity between the nucleotide sequences obtained and the data in the NCBI database. The level of similarity to cattle (*Bos taurus*) was 99%.

**Figure 3:**
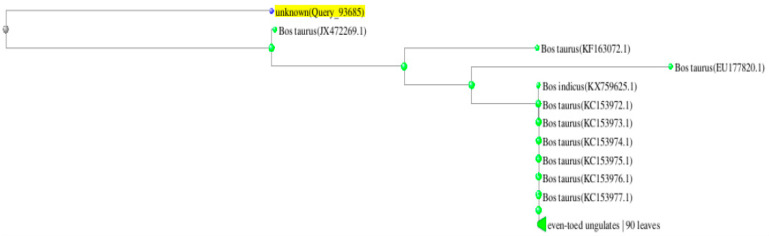
Phylogenetic tree obtained by the NJ method.

The sequence obtained was presented together with the sequences from the BLAST database, and a phylogenetic tree was constructed by the neighbour-joining (NJ) method. Sequences from the database are described according to the species name and accession number (Figure 3).

No results were obtained for the other pairs of primers. This was probably due to DNA degradation and the small amount of DNA isolated.

The genetic analysis—sequencing of a fragment of cyt b and bioinformatics analysis—yielded a reliable result that could be used to formulate conclusions. The tissue fragment taken from the keychain showed 99% similarity to cattle (*Bos taurus*).

## 4. Discussion

Many universal primers used in species identification of vertebrates have been described. In the case of identification based on biological traces [4,29,30], degraded DNA can be expected. In this case, the use of primers with a large amplification product (above 500 bp) may be less effective [31]. Hajibabaei et al. developed primers for the cytochrome b gene, whose short sequences are suitable for samples with degraded DNA, maintaining an excellent ability to distinguish species [32,33]. The mitochondrial cyt b is widely used in forensic and taxonomic analyses [34]. This gene ensures very precise reconstruction of the phylogenesis of mammals at various taxonomic levels (order, family, genus, and species) [35].

Yang et al. drew attention to the problem of the use of materials from endangered species in cosmetic and medicinal products [36]. Molecular methods of DNA identification can be used for rapid detection of ingredients of illegal origin. This is a reference point for quality control and supervision of trade in traditional medicines of animal origin.

Analysis of mitochondrial DNA was used to determine the species origin of animal bones from excavations in Northern Nigeria and Northern Cameroon. Universal primers for the cyt b were used for the analysis. Positive identification was achieved in 5 of 14 cases; the material was confirmed to have come from sheep and goats [37]. In the case of degraded samples, in which DNA has undergone fractionation, universal primers which are highly effective at amplifying the target region are necessary. Analysis of mitochondrial DNA has become an essential tool for identification of species in routine practice, and a commonly employed marker is a fragment of the cyt b, using the L15601/H15748 primer pair. Lopez-Oceja et al. confirmed the effectiveness of this pair of primers for 63 animal species belonging to 38 families from 14 orders and five classes (Mammalia, Aves, Reptilia, Actinopterygii, and Malacostraca) [25]. The species could be determined in all cases, which confirms that the fragment analysed had high potential for species identification. Later studies confirmed the effectiveness of the same primers for seven species of cetaceans and 22 species of bats [38,39].

Other markers verifying species identity are mitochondrial ribosomal genes 12S and 16S. Boukhdoud et al. used faeces samples to determine animal species by amplifying a 12S rRNA gene fragment using primers L1085/H1259 [40]. Among 234 environmental samples of good quality, a DNA amplification product was obtained from 188 (80.34%), with an average sequence length from 175 to 195 bp for most samples. In this way, 12 species of wild animals, two species of domesticated mammals, three species of birds, and one reptile species were determined. The same pair of primers (L1085/H1259) was used to test samples of fresh and smoked meat [19]. On the basis of 219 meat samples, 34 animal species were identified. For 27.9% (61/219) of samples, discrepant results were obtained between the species indicated by the producer and the results of NCBI BLAST analysis.

Dalen et al. analysed species identity based on the bones of birds, using the gene encoding ribosomal subunit 16S rRNA [41]. This gene is present in all prokaryotic and eukaryotic organisms, and the degree of variation in its sequence increases with the phylogenetic distance between organisms. Among 25 samples tested, a reliable result was obtained for 18 fragments. The probability of agreement between the sequences obtained and the reference sequences was very high, ranging from 97.5% to even 100% [41].

Other studies have also used environmental samples in the form of faeces [22]. Primers L2513 and H2714, amplifying a fragment of the 16S rRNA gene, were used for amplification. An amplification product was obtained for 58.4% of samples, and a total of 12 different mammalian species were determined. The failure to obtain an amplification product may be explained by the lack of high-quality DNA from faeces samples that were not fresh. Since target cells in faeces, like all cells exposed to the elements, undergo degradation [42], fresh samples should be collected in the field, taking all precautions to avoid degradation. The same primers (L2513 and H2714) have also been used to verify the species of fish [43] and wild cats [21].

In the present study, no result was obtained for the 16S rRNA sequence, possibly due to the small amount of genetic material obtained or severe degradation of the sample.

In comparison with research by other authors, the fragment of the cyt b analysed in the study was short, but highly informative. Therefore, it can be used for species identification, including degraded samples, e.g., from bone. In general, the use of mini-barcodes of less than 200 bp is recommended for successful amplification [44]. Universal primers cyt b L15601/H15748, with a short amplicon of 148 bp, can be analysed to identify forensic samples, even if degradation of the sample is suspected.

This study demonstrates the wide application of identification based on molecular biology techniques and the effectiveness of this method in the case of highly degraded samples.

## 5. Conclusions

The material analysed was bone tissue, which was found to have a 99% probability of belonging to domestic cattle (*Bos taurus*). The animal species from which the keychain was made is not under protection by the provisions of the Habitats Directive or the Washington Convention [23,24]. Species identification by molecular methods is extremely important for analysing material when the species cannot be identified on the basis of morphological characteristics. Identification systems are still being improved to enable rapid determination of species from various types of samples. The methods presented have proven effective at identifying the species of an animal. The universal primers cyt b L15601/H15748 were shown to be useful for identifying the species of an animal on the basis of polished bone. It was demonstrated that for DNA from biological material which has been processed (refined), the species origin can be confirmed only on the basis of a short amplicon (148 bp).

## Figures and Tables

**Table 1:** Universal mtDNA primers used for species identification.

Locus	Forward	Sequence (5′–3′)	Reverse	Sequence (5′–3′)	Size (bp)	References
Cytochrome b	L15601	TACGCAATCCTACGATCAATTCC	H15748	GGTTGTCCTCCAATTCATGTT	148	[25]
Cytochrome b	L14841	CCATCCAACATCTCCGCATGATGAAA	H15149	CCCTCAGAATGATATTTGGCCTCA	306	[26]
Cytochrome b	L14553	CTACCATGAGGACAAATATC	MOUSE-TR	TTCCATTTYTGGTTTACAAGACCA	990	[27,28]
12S rRNA	L1085	CCCAAACTGGGATTAGATACCC	H1259	GTTTGCTGAAGATGGCGGTA	215	[17]
16S rRNA	L2513	GCCTGTTTACCAAAAACATCAC	H2714	CTCCATAGGGTCTTCTCGTCTT	244	[17]

## Data Availability

Data are contained within the article.

## References

[1] Giles R.E., Blanc H., Cann H.M., Wallace D.C. (1980). Maternal inheritance of human mitochondrial DNA. Proc. Natl. Acad. Sci. USA.

[2] Nakonieczna S., Grela M., Listos P., Gryzinska M. (2019). Molekularne metody identyfikacji gatunkowej wykorzystywane w ekspertyzach sądowych. J. Anim. Sci. Biol..

[3] Dalvin S., Glover K.A., Sørvik A.G., Seliussen B.B., Taggart J.B. (2010). Forensic identification of severely degraded Atlantic salmon (*Salmo salar*) and rainbow trout (*Oncorhynchus mykiss*) tissues. Investig Genet..

[4] Herbert P.D.N., Stoeckle M.Y., Zemlak TSFrancis C.M. (2004). Identification of Birds through DNA Barcodes. PLoS Biol..

[5] Anderson S., Bruijn M.H., Coulson A.R., Eperon I.C., Sanger F., Young I.G. (1982). Complete sequence of bovine mitochondrial DNA, conserved features of the mammalian mitochondrial genome. J. Mol. Biol..

[6] Xu X., Aranson U. (1994). The complete mitochondrial DNA sequence of the horse Equus caballus: Extensive heteroplasmy of the control region. Gene.

[7] Hiendleder S., Lewalski H., Wassmuth R., Janke A. (1998). The complete mitochondrial DNA sequence of the domestic sheep (*Ovis aries*) and comparison with the other major ovine haplotype. J. Mol. Evol..

[8] Ursing B.M., Arnason U. (1998). The complete mitochondrial DNA sequence of the pig (*Sus scrofa*). J. Mol. Evol..

[9] Luikart G., Gielly L., Excoffier L., Vigne J.D., Bouvet J., Taberlet P. (2001). Multiple maternal origins and weak phylogeographic structure in domestic goats. Proc. Natl. Acad. Sci. USA.

[10] Cooper A., Lalueza-Fox C., Anderson A., Rambaut A., Austin J., Ward R. (2001). Complete mitochondrial genome sequences of two extinct moas clarify ratite evolution. Nature.

[11] Haddrath O., Baker A.J. (2001). Complete mitochondrial DNA geonome sequences of extinct birds: Ratite phylogenetics and the vicariance biogeography hypothesis. Proc. Biol. Sci..

[12] Hofreiter M., Paijmans J.L., Goodchild H., Camilla F., Speller C.F., Barlow A., Fortes G.G., Thomas A., Ludwig A., Collins M.J. (2014). The future of ancient DNA: Technical advances and conceptual shifts. Bioessays.

[13] Skuza L., Demska K., Adamczyk A. (2015). Barkoding jako nowoczesne narzędzie biologii molekularnej. Postępy Biol. Komórki.

[14] Ajmal Ali M., Gyulai G., Hidvégi N., Kerti B., Al Hemaid F.M., Pandey A.K., Lee J. (2014). The changing epitome of species identification—DNA barcoding. Saudi J. Biol. Sci..

[15] Kowalczyk M., Zawadzka E., Szewczuk D., Gryzińska M., Jakubczak A. (2018). Molecular markers used in forensic genetics. Med. Sci. Law.

[16] Kelle J., Carmon J., Pucherelli S., Hosler D. (2014). Identification of Unknown Organisms by DNA Barcoding: A Molecular Method for Species Classification. Tech. Memo..

[17] Kitano T., Umetsu K., Tian W., Osawa M. (2007). Two universal primer setsfor species identification among vertebrates. Int. J. Legal Med..

[18] Wultsch C., Waits L.P., Kelly M.J. (2014). Noninvasive individual and species identification of jaguars (Panthera onca), pumas (*Puma concolor*) and ocelots (*Leopardus pardalis*) in Belize, Central America using cross-species microsatellites and faecal DNA. Mol. Ecol. Resour..

[19] Dell B.A., Masembe C., Gerhold R., Willcox A., Okafor C., Souza M. (2021). Species misidentification in local markets: Discrepancies between reporting and molecular identification of bushmeat species in northern Uganda. One Health.

[20] Boukhdoud L., Saliba C., Kahale R., Bou Dagher Kharrat M. (2021). Tracking mammals in a Lebanese protected area using environmental DNA-based approach. Environ. DNA.

[21] Angulo A.S., Fajardo F.E., Salom-Pérez R., Carazo-Salazar J., Taylor F., Pilé E., Quesada-Alvarado F., Blanco-Peña K. (2023). Identification of anthropogenic impact on natural habitats by antimicrobial resistance quantification in two neotropical wild cats and their geospatial analysis. J. Wildl. Dis..

[22] Yan P., Wu X., Yang R., Li X., Yang B. (2011). DNA-based species identification for faecal samples: An application on the mammalian survey in Mountain Huangshan Scenic Spot. Afr. J. Biotechnol..

[23] Council Directive 92/43/EEC of 21 May 1992 on the Conservation of Natural Habitats and of Wild Fauna and Flora. https://eur-lex.europa.eu/LexUriServ/LexUriServ.do?uri=CONSLEG:1992L0043:20070101:en:PDF.

[24] Convention on International Trade in Endangered Species of Wild Fauna and Flora (Signed at Washington, D.C., on 3 March 1973. https://cites.org/sites/default/files/eng/disc/CITES-Convention-EN.pdf.

[25] Lopez-Ocejaa A., Gamarraa D., Borraganb S., Jiménez-Morenoc S., de Pancorboa M.M. (2016). New cyt b gene universal primer set for forensic analysis. Forensic Sci. Int.-Genet..

[26] Kocher T.D., Thomas W.K., Meyer A., Edwards S.V., Paabo S., Villablanca F., Wilson A.C. (1989). Dynamics of mitochondrial DNA evolution in animals: Amplification and sequencing with conserved primers. Proc. Natl. Acad. Sci. USA.

[27] Sullivan J., Markert J.A., Kilpatrick C.W. (1997). Phylogeography and molecular systematics of the *Peromyscus aztecus* species group (Rodentia: Muridae) inferred using parsimony and likelihood. Syst. Biol..

[28] Wade N.L. (1999). Molecular Systematics of Neotropical Deer Mice of the *Peromyscus mexicanus* Species Group. Master’s Thesis.

[29] Hebert P.D., Cywinska A., Ball S.L. (2003). Biological identifications through DNA barcodes. Proc. Biol. Sci..

[30] Parson W., Pegoraro K., Niederstätter H., Föger M., Steinlechner M. (2000). Species identification by means of the cytochrome b gene. Int. J. Legal Med..

[31] Xie J., Zhu W., Zhou Y., Liu Z., Chen Y., Zhao Z. (2015). Identification of mammalian species using the short and highly variable regions of mitochondrial DNA. Mitochondrial DNA.

[32] Hajibabaei M., Smith M., Janzen D.H., Rodriguez J.J., Whitfield J.B., Hebert P.D. (2006). A minimalist barcode can identify a specimen whose DNA is degraded. Mol. Ecol. Notes.

[33] Hajibabaei M., Singer G.A., Clare E.L., Hebert P.D. (2007). Design and applicability of DNA arrays and DNA barcodes in biodiversity monitoring. BMC Biol..

[34] Hsieh H.M., Chiang H.L., Tsai L.C., Lai S.Y., Huang N.E., Linacre A., Lee J.C. (2001). Cytochrome b gene for species identification of the conservation animals. Forensic Sci. Int..

[35] Tobe S.S., Kitchener A.C., Linacre A.M. (2010). Reconstructing mammalian phylogenies: A detailed comparison of the cytochrome B and cytochrome oxidase subunit I mitochondrial genes. PLoS ONE.

[36] Yang F., Ding F., Chen H., He M., Zhu S., Ma X., Jang L., Li H. (2018). DNA Barcoding for the Identification and Authentication of Animal Species in Traditional Medicine. Evid. Based Complement. Altern. Med..

[37] Newman M., Canada J., Parboosingh S., Bridge P.J. (2002). Identification of Archaeological Animal Bone by PCR/DNA. J. Archaeol. Sci..

[38] Lopez-Ocejaa A., Lekubeb X., Ruizc L., Mujika-Alustizad J.A., De Pancorboa M.M. (2019). CYT B L15601 and H15748 primers for genetic identification of cetacean species. Forensic Sci. Int.-Genet..

[39] Arnaout Y., Djelouadji Z., Robardet E., Cappelle J., Cliquet F., Touzalin F., Jimenez G., Suzel Hurstel S., Borel C., Picard-Meyer E. (2022). Genetic identification of bat species for pathogen surveillance across France. PLoS ONE.

[40] Boukhdoud L., Saliba C., Parker L.D., McInerney N.R., Ishac Mouawad G., Kharrat M., Kahale R., Chahine T., Maldonado J.E., Bou Dagher Kharrat M. (2020). First DNA sequence reference library for mammals and plants of the Eastern Mediterranean Region. Genome.

[41] Dalén L., Lagerholm V.K., Nylander J.A.A., Barton N., Bochenski Z.M., Tomek T., Rudling D., Ericson P.G.P., Irestedt M., Stewart J.R. (2017). Identifying Bird Remains Using Ancient DNA Barcoding. Genes.

[42] Kohn M., Knauer F., Stoffella A., Schröder W., Pääbo S. (1995). Conservation genetics of the European brown bear—A study Rusing excremental PCR of nuclear and mitochondrial sequences. Mol. Ecol..

[43] Evans N.T., Olds B.P., Renshaw M.A., Turner C.R., Li Y., Jerde C.L., Mahon A.R., Pfrender M.E., Lamberti G.A., Lodge D.M. (2016). Quantification of mesocosm fish and amphibian species diversity via environmental DNA metabarcoding. Mol. Ecol. Resour..

[44] Schneider P.M., Bender K., Mayr W.R., Parson W., Hoste B., Decorte R., Cordonnier J., Vanek D., Morling N., Karjalainen M. (2004). STR analysis of artificially degraded DNA-results of a collaborative European exercise. Forensic Sci. Int..

